# The viral picture of well-being: Biggest concerns, losses, and unintended gifts of COVID-19 in the Philippines

**DOI:** 10.1371/journal.pone.0288058

**Published:** 2023-07-06

**Authors:** Ma. Teresa Tuason, Kelly Perniciaro, Richel Lamadrid, Jego Mallillin, C. Dominik Güss

**Affiliations:** 1 Department of Public Health, University of North Florida, Jacksonville, Florida, United States of America; 2 University Research and Innovation Center, St. Louis University, Baguio City, Philippines; 3 Ateneo de Manila University Quezon City, Quezon City, Philippines; 4 Department of Psychology, University of North Florida, Jacksonville, Florida, United States of America; Research, Training and Management International, BANGLADESH

## Abstract

The COVID-19 pandemic revealed more than anticipated about global human functioning and resiliency. This Philippines-based study replicated a recent U.S. COVID analysis on psychological well-being (PWB). Factors examined herein were grouped into categories for analysis: 1) predictors of PWB, 2) areas of greatest stress or worry (biggest concerns), 3) perceived or real losses across SES, and 4) identified “unintended gifts” across PWB. Participants (n = 1345) were volunteers who responded to an online survey from August to September 2021, peak of the Delta variant. Three general groups of predictors (biological, psychological, and socio-economic) contributed to PWB. A regression model containing a total of 11 variables was significant, *F*(11, 1092) = 116.02, *p* < .00, explaining 53.9% of the variance. The model indicated PWB was significantly predicted by physical health, age, spirituality, emotional loneliness, social loneliness, sense of agency, and income. The strongest predictors of PWB were a sense of agency, social loneliness, and spirituality. Qualitative data analysis was conducted examining biggest concerns, losses due to COVID, and unintended gifts. Top ranking participant concerns were the health of family and friends, personal wellness, and governmental inefficiency/lack of concern. Losses compared to pre-COVID life were analyzed by SES group, with the most frequent responses being missing face-to-face interactions and the freedom to go/do what they please. Low SES groups were most likely to endorse missing everyday routine and experiencing changes in housing conditions due to the pandemic. Unintended gifts of COVID explored by PWB, high PWB individuals significantly appreciated intentional time with family and friends, deepening their spiritual lives, the ability to work from home, less pollution, and more time for physical exercise. Low PWB individuals reported nothing gained, except more time playing video games and watching TV. Those with higher PWB identified more unintended gifts of COVID and coped more actively.

## Introduction

Developing countries experienced greater hardships than developed nations during the COVID pandemic. COVID highlighted disparities such as limited resources to test and track COVID, minimal laboratory systems with protocol safety measures, sparse medical supplies, and less qualified healthcare workers which raise mortality rates [1]. Research showed in 2019 that 55% of global communities, mainly developing countries, had no social protection which exacerbated economic disparities leading to food insecurity, limitations to services, and strain on agriculture economics during COVID [2]. These strains also contributed to less availability to education and reduced access to health insurance [3]. By having the highest reported cases of COVID in Southwest Asia and higher confirmed COVID cases and death per capita when compared to other countries in Asia, the Philippines stands out as a developing country recovering from the devastation of COVID [4]. With its high population density [5], and its high poverty rate (23.7%) in the first semester of 2021 [6], the majority of Filipinos were vulnerable to COVID-related disparities.

Given these consequences of COVID in the Philippines, and being mindful of the disparities in resources, this study has two main objectives: 1) to identify the predictors of psychological well-being, among biological, psychological, and socio-economic variables, as previously conducted in the U.S. [7], using the biopsychosocial model [8]; and 2) to investigate the biggest concerns, losses, and unintended gifts of COVID in a developing country. The current study was conducted during the peak of the Delta Variant (SARS-CoV-2) while still on lockdown from August 2021-September 2021.

### Philippines’ contracting covid

On March 12, 2020, then-Philippine president Rodrigo Duterte ordered a lockdown–officially referred to as a “community quarantine” [9]–imposed upon the country’s National Capital Region (NCR) [10], which then lasted up to September 2021. The Philippines recorded its first case of the novel coronavirus from a 38-year-old woman from Wuhan, China [11], and a week after, the Philippines recorded its first case of local transmission of the virus from a 62-year-old man from San Juan, a city within NCR [12]. When a 68-year-old man from Butuan City, Agusan del Norte (Region XIII), was confirmed to have tested positive, this marked the moment that COVID-19 had spread in all 17 regions of the Philippines [13]. In the Philippines at around that time (August 2021), there were 125,470 cases of COVID and 1,054 COVID deaths, and cumulatively, 2,060,965 cases and 34,062 deaths [14], which then skyrocketed at the end of the following month with 2,549,966 cases and 38,294 deaths [15]. The Delta variant added more stress to healthcare programs and increased intensive care unit (ICU) occupancy, while 76% of ICU beds were taken in Metro Manilla [16].

The rise in cases and deaths are all despite the fact that the Philippine government responded with the harshest and prolonged lockdowns with strong military and police enforcement of wearing masks and social distancing through arrests and fines [17]. Although Filipinos lamented the impracticality of purchasing and using face shields [18], the Philippine administration asserted the science that backed up how face shields can lower the virus’s transmission risk transmission, quoting a Lancet study and various numbers [19]. In addition, communities in the Philippines were deprived of equitable access to resources and subjected to overbearing government policies. Even as COVID-19 vaccines have rolled out, areas of the Philippines continually have gone through multiple types of quarantines: the Enhanced Community Quarantine (ECQ) prohibited group gatherings, public transportation, school attendance, travel for land, sea, and air, and the heavy reinforcement of quarantining at home [20], while the General Community Quarantine (GCQ) provided more relaxed restrictions in given areas [21]. Though ECQ and GCQ were implemented, Metro Manila’s GCQ had five alert levels, from 1 to 5, with movement restrictions being stricter as the alert level number increased [22].

The World Health Organization tried to promote access to vaccines for lower income countries with the COVID-19 Vaccines Global Access (COVAX) but was ineffective due to countries’ insufficient management of vaccines, reduced healthcare providers and facilities, and limited information to communities [23]. Rates of people accepting COVID vaccines are lower in developing countries since there is less awareness about COVID risks and the effectiveness of vaccines [24], including in the Philippines [25]. Even international pundits noticed the Philippine government’s shortcomings in the country’s recovery. According to *Nikkei*’s COVID-19 Recovery Index—a monthly ranking of qualified countries depending on “infection management, vaccine rollouts, and social mobility,”—as of September 30, 2021, the Philippines ranked dead last at 121^st^ overall [26].

### Coping during covid-19

Research shows that those in the Philippines adhered to COVID protocols in order to cope during turbulent times [27, 28]. According to Bawingan et al. [28], Philippine residents coped with their physical health and mental health by doing leisure activities, communicating with others, and restricting the amount of time watching COVID news [28]. Callueng et al. identified measurements of resiliency and found that higher national resiliency in older Filipinos with religiosity and conservative political stances, and lower national resilience in those high distress and threat perception [29]. Resiliency was also associated with mental health and life contentment for reducing stress related to COVID among college nursing students [30]. Researchers found that residents who experienced adjustments to community resources by quarantining had personal perspectives of hope and emotional wellness [31].

Extant research on students in higher education includes coping with recreational activities and working multiple jobs [32], and by limiting in-person meetings [33]. Students who identified with more coping skills and high levels of individual resiliency had less exhaustion from lockdown mandates [34], while the lack of internet access showed lower ratings of taking care of oneself and less social connection [35]. Students from lower social economic families had less involvement in self-reflection activities, while most students engaged in being compassionate with themselves [35]. Additionally, teachers reported significant rates of distress and anxiety [27] and coped by devoting themselves to work and using “active” and “passive” coping strategies [36].

Religious and spiritual coping such as “dungaw” (having statues of Mary or the saints shown in their windows) was used during COVID foster connection within the church community [37], enhance spiritual rituals, and communicate with family members [38, 39]. Coincidently, mothers with increased spirituality had lower depression and anxiety about violence in the community [40] and greater personal well-being [41]. Utilizing spirituality as a means of coping allows connection to cultural values and enhances psychological wellbeing.

Regarding coping with natural disasters in the Philippines, social media is often used to connect with social support and document experience with photos and videos to process emotions [42]. Docena found that resiliency in the community decreased anxiety for dislocated survivors [43], while other typhoon survivors blamed themselves and restricted positive thoughts about their circumstances [44].

Overall, coping with COVID in the Philippines shows how coping relates to socio-demographic factors, cultural values, and disparities in their circumstances. Thus, the current study investigates the predictors of psychological well-being among biological (gender, age, physical health), psychological (spirituality, emotional loneliness, social loneliness, sense of agency), and socio-economic variables (hours communicating on social media, neighborhood safety, job security, income), to replicate the U.S. study [7] and identify the similarities and differences between the two countries. More importantly, the study explores how Filipinos are coping and connecting to the unintended gifts during the pandemic, while defining losses and the biggest concerns people face due to COVID.

## Materials and methods

### Procedure

Concerned with the impact of COVID in the Philippines, together with colleagues from the country, we gathered data during the peak of the COVID-19 Delta variant, August to September 2021. We replicated a study that we had conducted in the U.S. [7], to be able to compare and investigate the conditions between the two. The study was approved by the University of North Florida Institutional Review Board with approval #1589677–7 (Coronavirus Survey 1).

Through a Qualtrics link, the 40-question survey was briefly introduced on social media websites as a “research study to gain a deeper understanding of how individuals experience the current crisis (in the Philippines) in a country with strict lockdowns but with rising COVID cases”. The survey link was posted on internet sites such as Facebook and specifically asked for volunteers who live in the Philippines. The Qualtrics link was also sent to published email addresses of professors and students in various universities all over the country. In addition, this link was also sent through a research data-collecting mechanism of a university in the northern Philippines, which was blasted by email to 23,000 students. After giving their written consent through clicking the response option in the Qualtrics link, participants responded to the survey, which took around 12 minutes to complete. Participants were not compensated and included only those 18 years and older.

### Participants

There were 1,605 Filipinos who responded to a survey through Qualtrics at the height of the Delta variant COVID pandemic while the country was on a strict lockdown and when vaccines were not as available in the country. Of the 1605 responses in the Qualtrics data file, the data went through several layers of cleaning that resulted in 1,345 participant responses used in the data analysis. The participants’ age ranged from 18 to 89 (*M* = 32.11, *SD* = 16.68, *N* = 1,213). Regarding gender, out of 1,345 Filipinos, 910 (67.7%) were female, 307 (22.8%) were male, 1 (0.1%) was transgender, 12 (0.9%) were gender non-conforming, 2 (0.1%) identified as “Other,” 13 (1.0%) preferred not to answer, and 100 (7.4) did not answer this question. Regarding ethnicity, of those 1,345 who indicated being of Filipino nationality, 1,075 (79.9%) selected Filipino ethnicity, 88 (6.5%) were Chinese Filipino, 48 (3.6%) were Spanish Filipino, 9 (0.7%) were Filipino-American, 17 (1.3%) selected “Other”, and 108 (8.0%) did not respond to this question.

In terms of cleaning the data, first we checked for straightliners, those whose calculated standard deviation for the survey of 8 PWB questions and for the survey of 6 sense of agency questions were zero. We removed 42 participants who were straightliners and had a *sd* = 0 for both surveys. Second, the PWB survey had a check question, which ended up being confusing for some participants, so we removed this question in the data analysis. Third, we decided to remove 171 participants who took less than 240 seconds (4 minutes) to respond to the survey to maintain the integrity of the responses. We then also removed 19 participants whose progress was slow and who responded to less than 20% of the entire survey. We also removed 18 participants who indicated that they were not Filipinos or were from a non-Filipino ethnic group. Lastly, we removed 10 participants who consented to the study but were 17 years old and not 18 and older. The resulting sample size is 1,345 participants, which comprises 83.8% of the original Qualtrics data of 1,605.

### Instruments

This study is a replication of a previous study on COVID in the U.S. [7], and as such, the instruments we used are identical to the previous study. We decided to use survey questionnaires that had good psychometric properties (reliability and validity in previous studies) and were brief. Additionally, we followed the common guidelines in creating surveys for the internet [45].

#### Psychological well-being

We assessed PWB with an 8-item scale on a 7-point Likert scale ranging from “strongly agree” to “strongly disagree,” where each of the items is linked to a construct on well-being studied in previous research: meaning and purpose, supportive and rewarding relationships, engaged and interested, contribute to the well-being of others, competency, self-acceptance, optimism, and being respected [46]. An overall mean score was calculated, with higher scores signifying higher PWB. The Cronbach’s alpha reliability of PWB in this study was .885.

To increase convergence with the PWB, we also included a subjective item for overall mental health which is a slider question from 0 (“worst you have ever been”) to 10 (“best you have ever been”) based on research [47]: “Thinking of the last week, how is your overall well-being? Select the point on the line that summarizes your overall well-being for the last week”. The correlation between the overall mean score of the 8-item scale and the one-item question was *r* = .556 (*N* = 1280), *p* < .001.

#### Biological variables

*Gender*. We asked participants’ gender with answer options as female, male, transgender, gender non-conforming, other, or prefer not to answer. For the hierarchical multiple regression analysis, we only included females (67.7%) and males (22.8%) as a dichotomous variable and coded the others together (2.1%) as missing values. 7.4% did not answer this question and were missing values.

*Age*. We asked for the participants’ age. As mentioned in the participants section, age ranged from 18 to 89 (*M* = 32.11, *SD* = 16.68, *N* = 1,213).

*Physical health*. We also included a subjective item for overall physical health, a slider question from 0 (“worst you have ever been”) to 10 (“best you have ever been”): “Thinking of the last week, how is your overall physical health? Mark the line below with an X at the point that summarizes your physical health for the last week”.

#### Psychological variables

*Spirituality*. We assessed spirituality with a slider question from 0 (“not at all”) to 10 (“very much”): “I consider myself to be a spiritual/religious person”.

*Emotional loneliness*. We assessed Emotional loneliness with the Loneliness Scale of De Jong Gierveld & Van Tilburg, which consisted of 3 items and an overall score ranging from 0 to 3, with three answer options of “no,” “more or less,” and “yes” [48]. For our purposes, “more or less” or “yes” was counted as 1. The survey has been applied successfully in seven other countries [49]. Cronbach’s alpha reliability was .567 for the emotional loneliness subscale.

*Social loneliness*. We assessed Social loneliness with the Loneliness Scale of De Jong Gierveld & Van Tilburg [48, 49] that consisted of 3 items and an overall score ranging from 0 to 3, with three answer options of “no”, “more or less”, and “yes”. For our purposes, “no” or “more or less” or was counted as 1. Higher scores depicted higher levels of loneliness. Cronbach’s alpha reliability was .819 for the social loneliness subscale.

*Sense of agency*. We used the Sense of Agency Scale of Oeldorf-Hirsch & Sundar that consisted of six items on a 9-point Likert scale from 1 - “not at all” to 9 - “a lot” [50]. An overall mean of the six items was calculated, higher mean scores denote a higher sense of agency. Cronbach’s alpha reliability was .896 for the overall scale.

#### Socio-economic variables

*Hours communicating on social media*. We assessed these hours with a slider question from 0 to 10 hours: “During an average day of the past week, how much time do you spend per average on social communication via smartphone, Facebook, and the many platforms?”

*Neighborhood safety*. We assessed neighborhood safety with a slider question from 0 (“not safe at all”) to 10 (“very safe”): “How safe would you say is your neighborhood?”

*Employment security*. We assessed current employment security with one question. Responses were coded as 1 –unemployed or looking for work, 2 –part-time work or uncertain (awaiting company decision, student, or uncertain self-employed), and 3 –in paid employment or retiree. Looking after family was coded as a missing value.

*Income*. We assessed income with a slider question from 0 (“with great difficulty”) to 10 (“very easily”): “Thinking of your household’s total monthly income NOW, is your household able to make ends meet *now*?”

#### Biggest concerns, losses, and unintended gifts related to COVID-19

We asked, “Currently, what are your biggest concerns? Please read through the list first and then select your 5 main concerns among the following:” 23 answer options gathered from a sample of 10 student volunteers in the U.S. were piloted to 5 Filipino collaborators. Based on their suggestions and edits, 20 answer options, including an “other” option for biggest concerns in the Philippines were used.

We asked, “Thinking of your daily routine now, what do you currently miss compared to your life a year ago before the coronavirus crisis? Please read through the list first and then select 5 things you miss among the following:” Ten answer options, including an “other” option, was given. The same 5 Filipino collaborators were consulted regarding the answer options.

We asked, “What are the unintended gifts of COVID for you? Please read through the list first and then select 5 things you miss among the following:” 24 answer options, including an “other” option was given. The same 5 Filipino collaborators were consulted regarding the answer options.

### Data analysis

Data were collected via a Qualtrics-link and downloaded in Excel. Further analyses were conducted using the IBM SPSS Statistics 26 software. First, we conducted Pearson correlation analyses among all variables and then a hierarchical regression with PWB as an outcome. Step 1 included the biological variables, step 2 the psychological variables, and step 3 the socio-economic variables. Additionally, we conducted and reported multiple regression analyses for the three groups of variables separately.

To analyze the qualitative data on identifying individuals’ coping strategies/ biggest concerns, what they missed, and the unintended benefits or gifts during COVID-19, we conducted frequency counts and percentages. For what individuals missed we conducted group comparison based on SES scores. For what individuals identified as unintended gifts of COVID-19, we conducted extreme group comparison based on PWB scores. For PWB comparisons, the lowest and highest tertiary groups were compared.

To determine minimum sample size, we run a power analysis in G*Power [51] for a medium effect size of f2 = 0.15 [52], an alpha level of 0.01, with a power of 0.95, and a total number of 11 predictors. The program showed a minimum sample size of 227, which was easily achieved.

## Results and discussion

### Hierarchical multiple regression predicting PWB

A hierarchical multiple regression was conducted with three groups of variables to predict PWB. Pearson correlations among variables are shown in Table 1. First, the three biological variables: gender, age, and physical health were entered. Second, the four psychological variables: spirituality, emotional loneliness, social loneliness, and sense of agency were added. Third, the four socio-economic variables: hours communicating on social media, neighborhood safety, employment security, and socio-economic status were added.

**Table 1:** Descriptive statistics and Pearson correlations for all variables.

	*M*	*SD*	PWB	Gender	Age	Phys H	Spirit	Emo L	Soc L	Sense of agency	Hrs. communicating	Neighborhood safety	Employment security
**PWB**	5.14	1.06											
**Gender**			-.02										
**Age**	32.11	16.68	.42***	.01									
**Phys H**	6.19	2.38	.44***	.03	.30***								
**Spirit**	6.99	2.49	.44***	-.09***	.31***	.32***							
**Emo L**	2.07	.90	-.38***	-.07*	-.44***	-.32***	-.18***						
**Soc L**	1.51	1.25	-.46***	.05	-.26***	-.26***	-.22***	.31***					
**S agency**	6.91	1.41	.60***	.02	.34***	.37***	.26***	-.33***	-.33***				
**Hrs on soc med**	5.06	3.02	-.08**	-.08**	-.26***	- .07**	-.01	.22***	.03	-.12***			
**Neighbor safe**	7.20	2.19	.32***	.04	.36***	.23***	.18***	-.28***	-.22***	.34***	-.17***		
**Job sec**	2.28	.48	.30***	.03	.49***	.19***	.18***	-.27***	-.20***	.24***	-.12***	.18***	
**SES**	6.49	2.24	.37***	.01	.36***	.25***	.16***	-.28***	-.24***	.36***	-.16***	.45***	.27***

Note. Gender 1 = female, 2 = male

* *p* < .05

** *p* < .01

*** *p* < .001.

PWB–psychological well-being; Phys H–physical health; Emo L- emotional loneliness; Soc L- social loneliness; S agency- Sense of agency; Hrs on Soc Med–Hours communicating on social media; Neighbor Safe- Neighborhood Safety; Job Sec- job security; SES- Socio-economic status

Preliminary analyses were conducted to ensure no violation of the assumptions of linearity, multicollinearity, normality, and homoscedasticity. For example, Pearson correlations (between -.46 and .60), tolerance (between .57 and .99), and VIF values (1.00 and 1.75) did not indicate multicollinearity.

The three biological variables alone explain 29.3% of the variance in PWB, *F*(3, 1181) = 163.50, *p* < .001. Gender, age, and physical health were significant predictors. Adding the four psychological variables significantly improved the model and explained 53.8% of the variance in PWB (an additional 24.5%), *F*change(4, 1168) = 154.76, *p* < .001. The effect size attributable to the addition of the psychological variables to the model, Cohen’s f^2^ = 0.25, indicates a large effect. The four psychological variables spirituality, emotional loneliness, social loneliness, and sense of agency were all significant predictors. The overall model with the 7 variables was significant, *F*(7, 1168) = 194.18, *p* < .001. When conducting regression analyses separately and only using the four psychological variables as predictors, they were also all significant predictors of PWB (*p*s < .001), *R*^2^ = .412, *F*(4, 1231) = 215.49, *p* < .001.

Adding the four social and economic variables in the third step further improved the model significantly and explained 53.9% of the variance in PWB (an additional 0.5%), *F*change(4, 1092) = 3.12, *p* = .01. The effect size attributable to the addition of the social and economic variables to the model, Cohen’s f^2^ = 0.05, indicates a small effect. Job security (*p* = .07), and socio-economic status (*p* = .02) were significant predictors. The overall model with the 11 variables was significant, *F*(11, 1092) = 116.02, *p* < .001. When conducting regression analyses separately and only using the four social and economic variables as predictors (communicating on social media, neighborhood safety, job security, and income), all were significant predictors of PWB (*p*s ≤ .001) except communicating on social media (*p* = .67), *R*^2^ = .193, *F*(4, 1157) = 69.16, *p* < .001.

Looking at the final model (see Table 2), among the biological variables, gender was not a significant predictor, but age and physical health were. All four psychological variables spirituality, emotional loneliness, social loneliness, and sense of agency were significant predictors. Among the four social and economic variables, hours communicating on social media, neighborhood safety, job security, and income only income was a significant predictor. Spirituality, sense of agency, and low social loneliness were the strongest predictors of psychological well-being.

**Table 2:** Hierarchical regression results for psychological well-being.

Variable	B	95% CI	for B	SE B	β	*R* ^2^	Δ*R*^2^
		*LL*	*UL*				
**Step 1: Biological variables**						.29***	
** Constant**	3.61	3.40	3.82	.11			
** Gender**	-.07	-.18	-.05	.06	-.03**		
** Age**	.02	.02	.02	.002	.31***		
** Physical health**	.16	.14	.18	.01	.36***		
**Step 2: Biological and psychological variables**						.54***	.24***
** Constant**	2.45	2.10	2.79	.17			
** Gender**	-.005	-.10	.09	.05	-.002		
** Age**	.007	.004	.01	.001	.11***		
** Physical health**	.06	.04	.08	.01	.14***		
** Spirituality**	.09	.07	.11	.01	.21***		
** Emotional loneliness**	-.08	-.13	-.02	.03	-.07**		
** Social loneliness**	-.18	-.22	-.14	.02	-.21***		
** Sense of agency**	.28	.24	.31	.02	.37***		
**Step 3: + socio-economic variables**						.54***	.01***
** Constant**	2.01	1.68	2.52	.22			
** Gender**	.01	-.09	.11	.05	.004		
** Age**	.005	.001	.008	.002	.07**		
** Physical health**	.06	.04	.08	.01	.13***		
** Spirituality**	.09	.07	.11	.01	.21***		
** Emotional loneliness**	-.08	-.13	-.02	.03	-.07**		
** Social loneliness**	-.18	-.22	-.14	.02	-.21***		
** Sense of agency**	.27	.23	.31	.02	.35***		
** Hours communicating social media**	.01	-.004	.03	.01	.03		
** Neighborhood safety**	-.001	-.02	.02	.01	-.001		
** Job security**	.10	-.007	.20	.06	.04		
** Income**	.03	.005	.05	.01	.06*		

Note. CI = BC Confidence interval, LL = lower limit, UL = upper limit. Gender was coded as 1 = female and 2 = male.

* *p* < .05

** *p* < .01

*** *p* ≤ .001

### Qualitative data results

#### Biggest concerns

Among 20 statements of people’s possible concerns during COVID-19, individuals were asked to identify five that resonated with them the most. The five biggest concerns in rank order of the most mentioned to the least are, “The health of family and friends contracting COVID”, “My own health”, “The government’s inefficiency and lack of concern for the people”, “The uncertainty of the entire situation”, “Healthcare professionals and medical communities in extreme stress”, “The economy crashing”, and “Limited vaccine roll out”. The concerns mentioned the least, of all 20 statements are “Having to continue to work because my job cannot be done remotely”, and “Not being able to pay rent”. The frequency count and percentages of each of the biggest concerns are in Table 3.

**Table 3:** Frequencies for biggest concerns.

Variable	Yes (*N out of 1*,*371*)	
	n	*%*
The health of family and friends (e.g., contracting COVID)	1193	87.0
My own health	886	64.6
The government’s inefficiency and lack of concern for the people	849	61.9
The uncertainty of the entire situation	729	53.2
Healthcare professionals and medical communities in extreme stress	721	52.6
The economy crashing	412	30.1
Limited vaccine roll-out	381	27.8
Other people not doing social distancing	364	26.5
Accidentally infecting others	321	23.4
Working/ going to school remotely	254	18.5
Possible social unrest or chaos	250	18.2
Prices of good increasing	208	15.2
My children’s school, future, health	189	13.8
My current living situation (e.g., living alone and feeling isolated, living with other people and not getting along)	178	13.0
Not having enough supplies (e.g., food, medical, hygiene) for my everyday	138	10.1
Losing my job and not having income	112	8.2
Not having enough supplies in my community	79	5.8
Having to continue to work because my job cannot be done remotely	35	2.6
Not being able to pay rent	33	2.4
Other	59	4.3

#### Losses due to COVID, what is missed

Among 10 statements of what people miss during COVID-19, including one which was open-ended and they could fill in, participants were asked to think of their daily routine and identify five that they missed the most, compared to pre-COVID life. We then conducted group comparison of what people missed, based on the subjective indicator of their socio-economic status (1–4 low, 5–7 middle, 8–10 high). For these frequency comparisons, the different SES groups were compared, and chi-square analyses were conducted. For these chi square comparisons and significance levels, we used Bonferroni-adjusted p values of .05/10 items, which equals .005. Thereby, the chi square significance of .001 suffices.

A chi-square test of independence was calculated comparing the frequency of missing “My usual routine: going to work/school” for the different SES groups, and a significant interaction was found (*χ*^2^ (2) = 49.32 *p* < .001). The low SES group were most likely to miss their usual routine than the middle SES group or the high SES group. Another chi-square test of independence was calculated comparing the frequency of missing “Housing conditions: where I lived before the crisis started” for the different SES groups, and a significant interaction was found (*χ*^2^ (2) = 30.13 *p* < .001). The low SES group were most likely to have missed something of their housing conditions compared to pre-COVID than the middle SES group or the high SES group.

No significant relationships were found, *p* > .05, between the SES groups in the following losses (in order of those rated more highly by all to least in all groups): missing “Face to face interactions with people”, “My freedom to go and do whatever I want”, “Certainty, health, and security: not being stressed about being sick”, “Going for leisure activities: restaurants, bars, shopping, beauty salon”, “Milestone celebrations (e.g., birthdays, weddings, graduations)”, and “Giving and receiving hugs”. What most people missed during COVID-19 and their SES appear to be independent from each other. The frequencies and Chi-square results of what is missed in the three SES groups are in Table 4.

**Table 4:** Frequencies and chi-square results for “What do I miss?” in low, medium, and high SES groups.

Variable	Low SES (*n* = 160)	15.1%	Medium SES (*n* = 644)	60.6%	High PWB (*n* = 258)	24.3%	
	n	%	n	%	n	%	χ^2^ (2)
My freedom to go and do whatever I want	125	78.1	515	80.0	212	82.2	1.09
Face to face interactions with people	127	79.4	523	81.2	213	82.6	.66
Certainty, health, and security: not being stressed about getting sick	102	63.7	438	68.0	166	64.3	1.74
Housing conditions, where I lived before the crisis started	24	15.0	36	5.6	5	1.9	30.13***
My usual routine: going to work/ school	110	68.8	384	59.6	97	37.6	49.32***
I miss giving hugs/ receiving them	64	40.0	236	36.6	117	45.3	5.89
Going for leisure activities: restaurants, bars, shopping, beauty salon	103	64.4	402	62.4	178	69.0	3.47
Going for medical check-ups without fear of getting COVID	46	28.7	213	33.1	103	39.9	6.23
Missing milestone celebrations (e.g., birthdays, weddings, graduations	105	65.6	415	64.4	165	64.0	0.12
Other _______	9	5.6	41	6.4	25	9.7	3.69

* *p* < .05

** *p* < .01

*** *p* < .001.

*p* = .05/10 items = .005

#### Unintended gifts

Among 24 statements of the unintended gifts during COVID-19, including one which was open-ended and they could fill in, participants were asked to think of their daily routine and what they gained, compared to pre-COVID life. We then conducted group comparison of what people gained and how they coped, based on their PWB (0–4.74 = low; 4.75–5.74 = middle, 5.75–7.1 = high). For what individuals identified as unintended gifts of COVID, we conducted extreme group comparison based on PWB scores, the lowest and highest tertiary groups were compared, and chi-square analyses were conducted. For these chi square comparisons and significance levels, we used Bonferroni-adjusted p values of .05/24 items, which equals .002. Thereby, the chi square significance of .001 suffices.

Chi-square tests of independence were calculated comparing the frequency of the unintended gifts of COVID and significant interactions were found for the following unintended gifts, with the high PWB group significantly identifying these more compared to the low PWB group: having more time with family and friends, having a richer spiritual/inner life, ability to work from home, better air quality/having less pollution, having more time for physical exercise, not having to drive/commute, avoid traffic, and having more time for myself to rest/reflect/re-energize.

Additionally, chi-square tests of independence were calculated comparing the frequency of the unintended gifts of COVID and significant interactions were found for the following unintended gifts, with the low PWB group significantly identifying these more compared to the high PWB group: nothing, getting to spend more time playing video games, getting to spend more time watching TV shows/movies, and having fewer responsibilities.

No significant relationships were found, *p* > .05, between the PWB groups in the following unintended gifts of COVID (in order of those rated more highly to least in both PWB groups): “Not spending a lot of money”, “Getting to spend more time with my pet”, “More time for hobbies/entertainment: gardening, baking”, “More time for daily living: cooking, cleaning, organizing”, “Having efficient working technology to continue work/be in school”, “Having more time in social media” and “Being creative and finding new ways to have fun”, among others. The frequencies and Chi-square results of the unintended gifts of COVID in the three PWB groups are in Table 5.

**Table 5:** Frequencies and chi-square results for “Unintended gifts” in low, medium, and high PWB groups.

Variable	Low PWB (*n* = 441)		Medium PWB (*n* = 414)		High PWB (*n* = 453)		
	n	%			n	%	χ2 (1)
Having more time with family or people I live with	226	51.2	280	67.6	355	78.4	73.94***
Having more time on social media/ Non-face-to-face-communication	91	20.6	77	18.6	57	12.6	11.00**
My current housing situation	24	5.4	31	7.5	29	6.4	1.49
Having more time for myself to rest/reflect/re-energize/slow down	183	41.5	202	48.8	241	53.2	12.49** (p = .002)
Not having to drive/commute so much and avoiding many hours in traffic	116	26.3	141	34.1	169	37.3	12.93** (p = .002)
Better air quality, having less pollution	108	24.5	105	25.4	167	36.9	20.61***
More time with daily living: cooking, cleaning, organizing	90	20.4	111	26.8	113	24.9	5.13
Getting to spend more time playing video games	85	19.3	43	10.4	18	4.0	53.13***
Getting to spend more time watching TV shows/movies	136	30.8	108	26.1	68	15.0	32.49***
Getting to spend more time relaxing with my pet	58	13.2	53	12.8	34	7.5	9.04
Getting projects done around the house	47	10.7	49	11.8	71	15.7	5.52
I’m not spending a lot of money	164	37.2	128	30.9	138	30.5	5.63
Having fewer responsibilities	50	11.3	25	6.0	19	4.2	18.29***
Having efficient working technology to continue work/be in school	67	15.2	78	18.8	97	21.4	5.78
Ability to work from home	68	15.4	97	23.4	132	29.1	24.15***
Having more time to catch up on work /schoolwork	80	18.1	63	15.2	58	12.8	4.91
Since I work in an essential workplace, more time to work and make money	5	1.1	9	2.2	19	4.2	8.81
Having more time for hobbies/ entertainment (e.g., gardening, boardgames, baking)	132	29.9	138	33.3	121	26.7	4.53
Being creative and finding new ways to have fun	69	15.6	75	18.1	74	16.3	0.99
Having more time for exercise and physical activity	61	13.8	83	20.0	115	25.4	18.81***
Having a richer spiritual/inner life	58	13.2	101	24.4	150	33.1	49.55***
The outdoors, nature, the environment	61	13.8	51	12.3	73	16.1	2.62
Nothing	49	11.1	9	2.2	6	1.3	55.62***
Other	10	2.3	9	2.2	13	2.9	0.53

** *p* < .002

*** *p* < .001.

*p* = .05/24 items = .002

## Discussion

The main goals of the study are twofold: first, to investigate the predictors of PWB during the COVID-19 pandemic in the Philippines and compare these to a similar study conducted in the U.S. [7], and second, to explore what Filipinos report as their biggest concerns, what losses they have missed due to COVID, and how they have coped or the unintended gifts they have discovered to do.

The overall regression model, which explains 53.9% of the variance, identifies seven significant predictors for PWB during the 2020 pandemic of COVID-19 in the Philippines as: physical health, age, spirituality, emotional loneliness, sense of agency, social loneliness, and income. The most significant predictors are a sense of agency, social loneliness, and spirituality. These findings of PWB’s predictors are similar yet different from that found in the U.S. [7]. Like the current findings, the U.S. study offers support for the biopsychosocial model [8, 53, 54]; however, the amount of variance contributed by each group of variables to the overall model is different. Our findings underscore the biological variables having the greatest portion of the variance (29.3%), with physical health and age being at the fore, while the U.S. findings underscore the psychological variables having the greatest portion of the variance. Moreover, the overall model in the U.S., explains 53.3% of the variance, which is slightly less than that of the Philippines (53.9%), and identifies six significant predictors for PWB as: physical health, spirituality, emotional loneliness, sense of agency, social loneliness, and employment security; while the Philippine predictors have two more significant ones which are age and income, but not employment security. These two additional predictors stress the importance of the Philippine context with prolonged lockdown and a high poverty rate [6], and thus age and income become key ingredients in PWB. Although both the Philippine and U.S. [7] findings have the most significant predictors of the overall model being psychological variables, such as a sense of agency and social loneliness, it is only in the Philippines that spirituality is one of the most significant. In the Philippines, religion and spirituality have been found to help with coping in decreasing depression and anxiety, especially in dealing with violent circumstances [40], with coping for people who are internally displaced [39], and with coping during COVID-19 even in ways that are not face-to-face but foster connection within the church community [37].

The biggest concerns reported by Filipinos during the height of the pandemic were the health of family and friends contracting COVID, their own health, and the government’s inefficiency and lack of concern to name a few. The concern about the health of family and friends before their own underscores the collective nature of the Philippines, which is also supported in honoring cultural standards such as sharing in hardship *pagdadamayan*, nurturing others *pagkalinga*, and helping each other with a communal goal *bayanihan* during COVID-19 [37]. The concern voiced out as the government’s inefficiency and lack of concern is validated by the fact that in *Nikkei’s* COVID-19 Recovery Index, the Philippines ranked last in terms of managing infection, rolling out vaccines and being socially mobile [26].

In terms of what is experienced as a loss, across SES groups, face-to-face interactions with people and having the freedom to go and do whatever they please, were the highest reported. These losses highlight the conditions of the lockdown with strict prohibitions for public transportation, school attendance, and travel by land, sea, and air [20], that people missed having the freedom to go and do what they want, and go out to places for leisure. The Philippines had the strictest lockdowns [17], people missed face-to-face interactions and celebrating milestone celebrations which previously reinforced interpersonal connections. When comparing individuals from low, middle, and high SES, the low SES group identified that they missed their usual routine of work/school and missed their housing condition previous to the pandemic, significantly more than the middle or high SES groups. These findings elucidate COVID’s impact on the people experiencing poverty, in that those in the low SES miss their usual everyday routine and that their housing conditions have changed. With the Philippines having such high population density [5] and a poverty rate of 23.71 [6], the consequences for the most vulnerable are felt daily.

When focusing on the unintended gifts of COVID, results show that there is more intentional time identified in the high PWB group such as: spending time with family and friends and having a richer spiritual/inner life, which are active coping with COVID that are identical to the findings conducted in the U.S. [7]. Such intentional coping is also seen in research studies in the Philippines that identify maintaining communication with family and friends, boosting a positive mindset [27], and doing enjoyable activities [32]. Having more time to enrich spiritual/inner life and having more time for oneself to rest/reflect/re-energize is also supported by coping that is researched by Cleofas in resting and being compassionate with oneself [35]. Additionally, having more time for physical exercise is also identified in previous research [28]. High PWB individuals identified many more unintended gifts of COVID, which also highlighted having a sense of agency in coping for emotional wellness and having a hope perspective [31]. On the other hand, low PWB individuals’ coping with using time in front of the screen are identical to the findings conducted in the U.S. [7]. The unintended gifts of COVID that were identified, despite differences in PWB, are not having to spend a lot of money and spending more time for daily living, to name a few. Such appreciation for having time for leisure activities is also supported by coping identified in the Philippines [28].

There are limitations of the current study which must be mentioned. First, although the sample size is quite large, the study is still a cross-section of the population of the Philippines at this one point in time. Secondly, we used an online survey and therefore had to restrict its length to lessen survey fatigue and to use several single items to assess constructs. Future research could use longitudinal designs to assess PWB throughout the pandemic in the Philippines.

Especially with the different COVID variants that are still present in the Philippines, important implications to coping with the protracted pandemic identify using intentional and active coping for promoting psychological well-being. The greater recognition of the vulnerabilities of the lower SES individuals, such as the shift in their everyday routine and fundamental changes to their housing conditions, will inform government and non-governmental agencies to decrease the health disparities in the pandemic.

## Conclusions

To sum up, biological, psychological, and socio-economic variables significantly predict psychological well-being in the Philippines during COVID—with a sense of agency, social loneliness, and spirituality as the most significant of the seven predictors. The socio-economic predictor of income highlights the conditions in developing countries such as the Philippines. The biggest concerns of individuals in the Philippines highlight concern for others over their own and worries that are exacerbated by the government’s inefficiency and lack of concern. The losses experienced during the pandemic have to do with the loss of freedom and the loss of face-to-face interaction, but unique to the low SES individuals are everyday routine and housing concerns. The unintended gifts that COVID brought, for those with high PWB, are the sense of agency of using time to deepen relationships with family and friends, with oneself, with God; attending to their physical and mental health, and appreciation of their environment. Our results reiterate the ingenuity and creativity of Filipinos to cope despite limited resources and circumstances—which expose the unintended lessons learned by the pandemic.
